# Training in robotic-assisted surgery: a systematic review of training modalities and objective and subjective assessment methods

**DOI:** 10.1007/s00464-024-10915-7

**Published:** 2024-05-30

**Authors:** A. Masie Rahimi, Ezgi Uluç, Sem F. Hardon, H. Jaap Bonjer, Donald L. van der Peet, Freek Daams

**Affiliations:** 1 Department of Surgery, Amsterdam UMC, Vrije Universiteit, Tafelbergweg 47, 1105 BD Amsterdam, The Netherlands; 2 Amsterdam Skills Centre for Health Sciences, Tafelbergweg 47, 1105 BD Amsterdam, The Netherlands; 3 Cancer Center Amsterdam, Amsterdam, The Netherlands

**Keywords:** Robotic-assisted surgery, Assessment methods, Robotic surgery training, Simulation training, Objective assessment, Subjective assessment

## Abstract

**Introduction:**

The variety of robotic surgery systems, training modalities, and assessment tools within robotic surgery training is extensive. This systematic review aimed to comprehensively overview different training modalities and assessment methods for teaching and assessing surgical skills in robotic surgery, with a specific focus on comparing objective and subjective assessment methods.

**Methods:**

A systematic review was conducted following the PRISMA guidelines. The electronic databases Pubmed, EMBASE, and Cochrane were searched from inception until February 1, 2022. Included studies consisted of robotic-assisted surgery training (e.g., box training, virtual reality training, cadaver training and animal tissue training) with an assessment method (objective or subjective), such as assessment forms, virtual reality scores, peer-to-peer feedback or time recording.

**Results:**

The search identified 1591 studies. After abstract screening and full-texts examination, 209 studies were identified that focused on robotic surgery training and included an assessment tool. The majority of the studies utilized the da Vinci Surgical System, with dry lab training being the most common approach, followed by the da Vinci Surgical Skills Simulator. The most frequently used assessment methods included simulator scoring system (e.g., dVSS score), and assessment forms (e.g., GEARS and OSATS).

**Conclusion:**

This systematic review provides an overview of training modalities and assessment methods in robotic-assisted surgery. Dry lab training on the da Vinci Surgical System and training on the da Vinci Skills Simulator are the predominant approaches. However, focused training on tissue handling, manipulation, and force interaction is lacking, despite the absence of haptic feedback. Future research should focus on developing universal objective assessment and feedback methods to address these limitations as the field continues to evolve.

**Supplementary Information:**

The online version contains supplementary material available at 10.1007/s00464-024-10915-7.

The rapid growth of robotic-assisted surgery over the last decade, driven by intraoperative technical benefits and, to a lesser extent, significant benefits for patients, has caused a demand for adequate surgeon training [1–5]. Robotic-assisted surgery is a complex minimally invasive surgery technique that requires a different training approach compared to open or laparoscopic surgery, where mentoring surgeons can directly teach through hands-on methodsh in a direct hands-on fashion [6, 7].

Simulation training has emerged as a valuable method for acquiring the technical skills needed for robotic surgery in a safe environment [8, 9]. Robotic simulation demonstrates a significant learning effect, with acquired skills proving transferable to actual robotic procedures [10–12]. Individual feedback during robotic simulation is essential for developing efficient learning curves [13, 14]. Moreover, assessing performance provides trainees and their supervisors with a clear representation of skill progress and the achievement of clinical proficiency [15].

In 2000, the da Vinci Surgical System, developed by Intuitive Surgical Inc., became the first FDA-approved robotic-assisted surgery (RAS) system that used a minimally invasive surgical approach [16]. Over the past two decades, various training modalities have been developed for the da Vinci Surgical System and other robotic systems, including didactic lectures, box training, virtual reality (VR) training, wet lab training involving either animal or human cadavers, proctoring, and mentoring [17]. Furthermore, a range of robotic skill assessment tools has been developed, categorized as subjective and objective tools [18, 19]. These tools vary from subjective assessment forms such as GEARS, R-OSATS and GOALS to objective built-in scoring systems [20]. Advances in computer processing have also facilitated the development of built-in scoring systems that collect objective data from robotic simulators [21].

The aim of this systematic review was to provide an overview of different training modalities and assessment methods for teaching and assessing surgical skills in robotic surgery. Specifically, the focus was on comparing the use of objective and subjective assessment methods.

## Methods

### Search strategy

A systematic review was conducted following the Preferred Reporting Items for Systematic Reviews and Meta-Analyses (PRISMA) guidelines [22]. The electronic clinical databases of Pubmed, EMBASE, and Cochrane were searched for original and primary articles from inception until March 1, 2023. The search consisted of a combination of free terms and database-specific index terms without applying any filters (Supplemental File A, Tables A1–A3). The search was limited to studies published in English.

All three electronic databases were independently consulted by two reviewers (M.R. & E.U.). Initially, the duplicate records of the three electronic databases were removed. Subsequently, a critical selection took place based on titles and abstracts. The full-text articles of the selected abstracts were critically studied and assessed for eligibility. If a full-text article was not available, the authors were contacted. Potential conflicts of inclusion between reviewers were discussed to reach a consensus. In case of discrepancies or disagreement regarding study inclusion, a third researcher (F.D.) was consulted.

### Inclusion criteria

Currently used training and assessment modalities for robotic-assisted surgery, regardless of the aim or specialty of the training/curriculum were identified. Included studies involved robotic-assisted surgery simulation training (such as box training, virtual reality training, cadaver training, animal tissue training, live animal training, and artificial tissue training) along with an assessment method (objective or subjective), including assessment forms, virtual reality scores, sensors, measuring systems, peer-to-peer feedback, or time recording. Reviews, letters, editorials, and comments were excluded. Studies without assessment methods (unsupervised training, no feedback, no assessments) or outcome measures were excluded.

### Data collection

To provide a comprehensible overview of the extensive range of robotic-assisted surgery training and assessments, the following data were collected per article: author, year of publication, title, training modality, assessment method, subjective/objective assessment, participants, and study findings.

Objective assessment was defined as an assessment expressed in objective values, such as time (s), motion (mm/cm), force (N), collisions, human performance (EEG, eye movement tracking, pupillary response, EMG), task-specific errors, clutch usage, and task trainer score (based on multiple objective parameters). Subjective assessment was defined as an assessment method not expressed in objective values, such as assessment forms (GEARS, OSATS, GOALS), video assessment feedback, or oral feedback (peer-to-peer or supervisor) after observation. Study data were collected and recorded in Microsoft Excel, and figures were created using GraphPad (Prism 9.0.0, San Diego, California USA).

### Data analysis

The data were analyzed using a narrative synthesis approach, which involved summarizing and synthesizing the results of the included studies in a narrative format. The results of the studies were grouped by the type of training modality (e.g., dry lab, virtual reality) and by subjective or objective outcome measure (e.g., subjective forms, time and motion measures, force measure).

### Basic and advanced courses

To illustrate the difference in the application of training modalities, the studies were divided into two groups: basic and advanced training. Basic courses encompassed fundamental skills, whereas advanced courses delved deeper into specific technical skills and procedural training. This distinction was made because the purpose of both training courses inherently impacts the training modality and, therefore, the assessment method. A novice robotic surgeon is more likely to begin with virtual training or dry lab, while a more advanced robotic surgeon is more likely to train in the wet lab with animal tissue models. Basic training aimed to introduce the trainee to the robotic surgery system, controls, user interface, camera, and to develop initial technical robotic surgery skills. Advanced training aimed to enhance pre-existing robotic surgery skills and apply them in high-fidelity models before applying the skills in practice.

#### Ethical committee review

As the study involved a systematic review focused on literature analysis, it was exempt from Ethical Committee review.

## Results

**Fig. 1:**
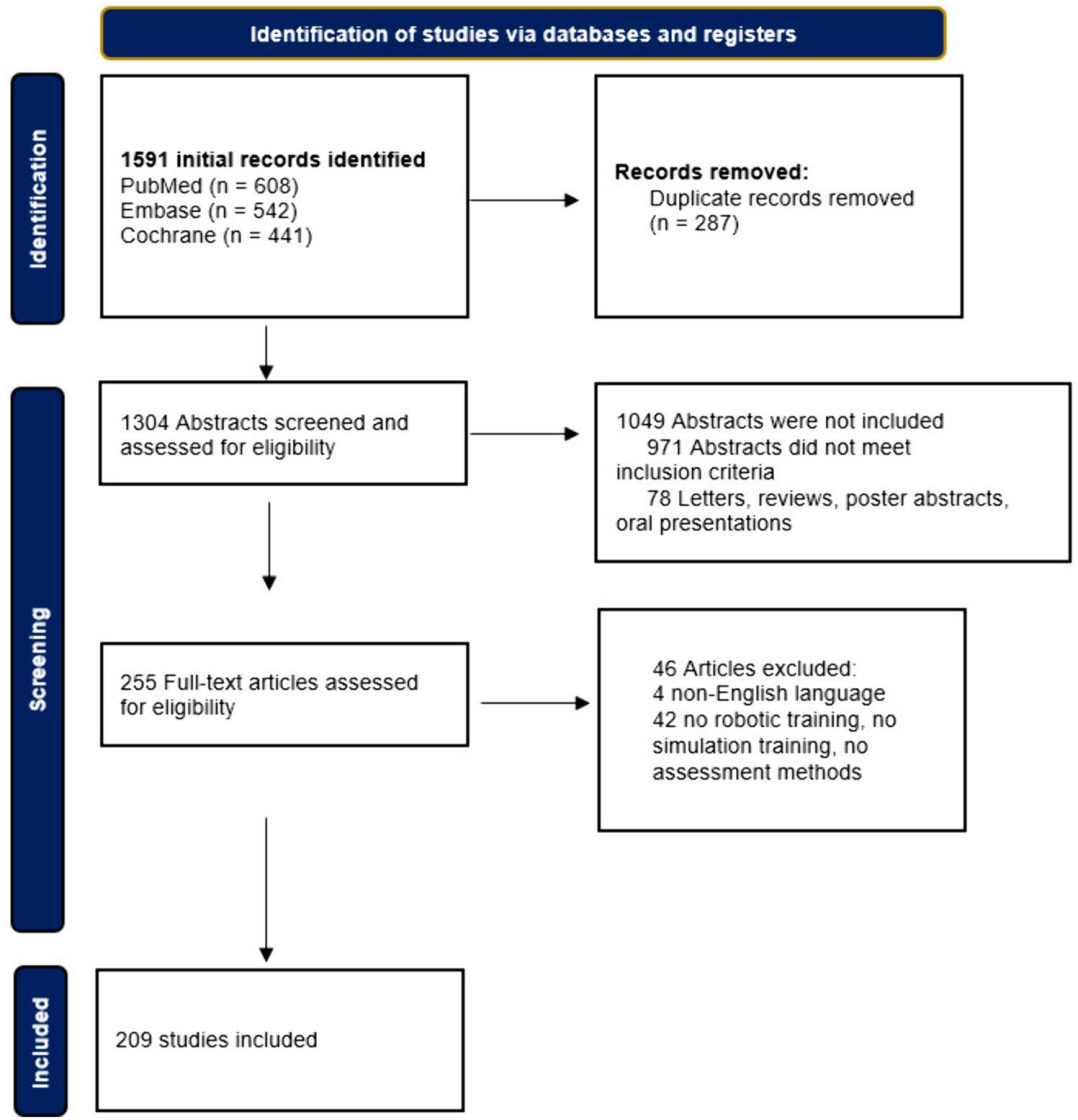
Flow diagram of study inclusions

The search yielded a total of 1591 studies. After abstract screening and examination of full-texts, 209 studies were identified that focused on robotic surgery training and included an assessment tool (Fig. 1). Multiple training modalities, assessment tools, and objective assessment parameters were identified.

### Training modalities

Three main training systems were identified in the studies: the da Vinci Surgical System was used in 91 studies with dry lab sessions and 30 studies with wet lab sessions, 36 studies using the dV-Trainer, and 64 studies utilized the da Vinci Surgical Skills simulator (Table 1). Some studies used a combination of training modalities to compare training outcomes or the effect on learning curves, such as a combination of virtual simulation and wet lab training, or a combination of dry lab training and hands-on cadaveric training.

**Table 1:** Study characteristics

Total studies	209
Total training modalities	255
da Vinci Surgical System	121 (121/255; 47%)
Dry lab	91
Wet lab	30
Animal models (porcine, chicken)	22
Cadaver training	2
Live procedure	6
dV-trainer Mimic Technologies	36 (14%)
da Vinci Surgical Skills simulator (dVSS)	64 (25%)
Other Robotic Training modalities	34 (13%)

N.B. The mentioned training modalities are based on the names used in the studies. Other Robotic Training modalities include: RobotiX Mentor, Senhance Surgical System, ProMIS and AdLap

### Basic and advanced courses

**Fig. 2:**
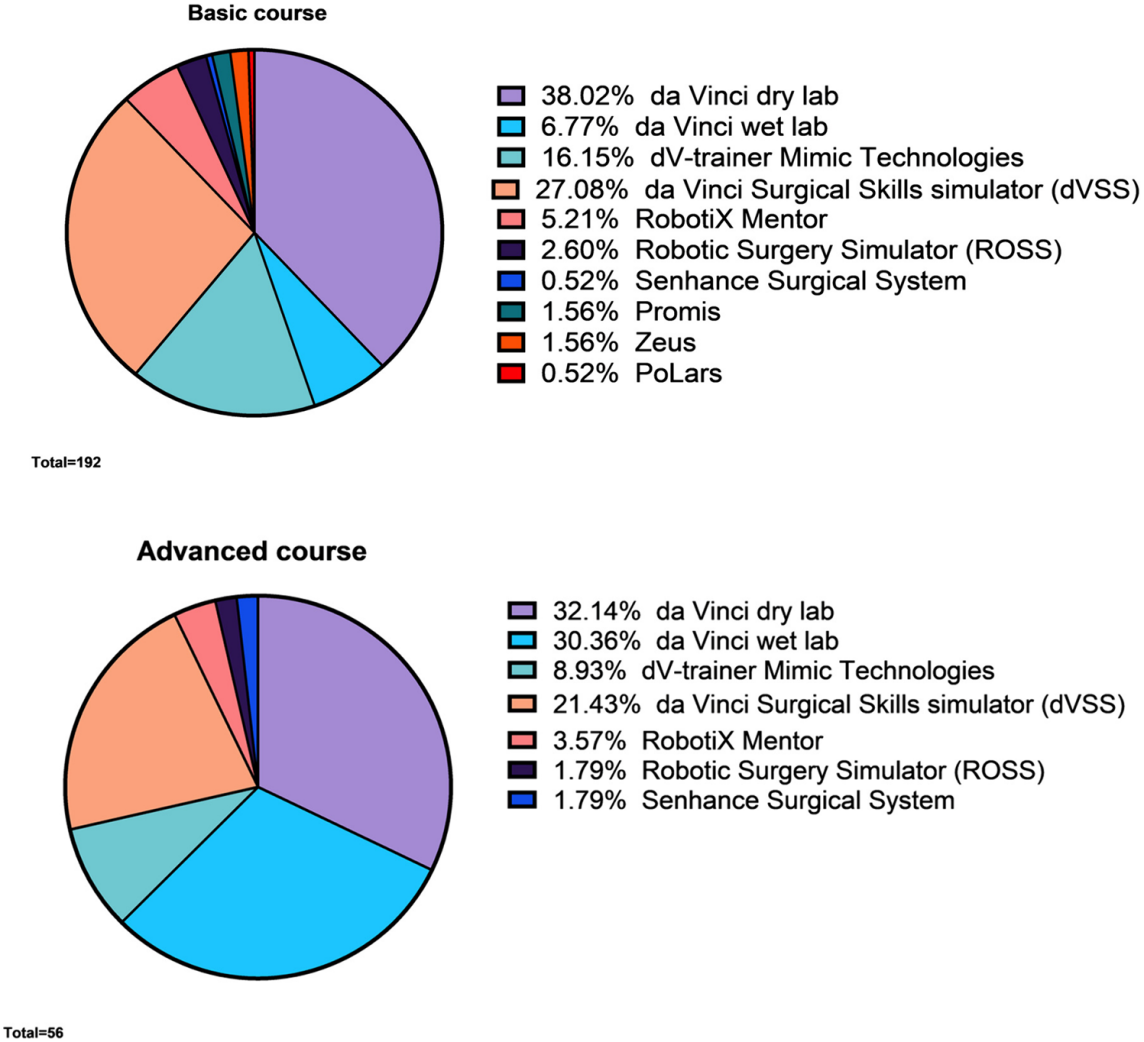
Basic and advanced modalities

Out of the 209 studies, 165 (79%) consisted of basic courses, and 44 (21%) consisted of advanced courses. The majority of the basic courses consisted of dry lab on the da Vinci Surgical System (73/195; 37%) and a virtual simulator (83/195; 43%) (Table 2, Fig. 2 and Supplemental File A, Fig. A1). A significant proportion (*n* = 42) of the basic course studies involved medical students (42/165; 25%), comparing learning curves of novices between different training modalities. In 28 studies (28/165; 17%), the training group consisted exclusively of residents, in 21 studies (21/165; 13%) of experts (surgeons, urologists, and gynaecologists), and in 74 studies (74/165; 45%) there was a combination of different experience groups (novices, intermediates and experts).

**Table 2:** Basic course characteristics

Total studies	165 (165/209; 79%)
Total training modalities	195
da Vinci Surgical System	86 (86/195; 44%)
Dry lab	73
Wet lab	13
Animal models (porcine, chicken)	12
Cadaver training	1
dV-trainer Mimic Technologies	31 (16%)
da Vinci Surgical Skills simulator (dVSS)	52 (27%)
Other Robotic Training modalities	26 (13%)

N.B. Other Robotic Training modalities include: RobotiX Mentor, Senhance Surgical System, ProMIS and AdLap

Regarding advanced courses, the largest training modality consisted of da Vinci dry lab training (18/60; 30%) and da Vinci wet lab training (17/60; 28%) on the da Vinci Surgical System (Table 3). Out of the 44 studies, 9 studies (9/44; 20%) had a training group consisting only of residents, 21 studies (21/44; 48%) had experts as participants, and 14 studies (14/44; 32%) had a combination of different experience groups.

**Table 3:** Advanced course characteristics

Total studies	44 (44/209; 21%)
Total training modalities	60
da Vinci Surgical System	35 (35/60; 58%)
Dry lab	18
Wet lab	17
Porcine models	10
Cadaver training	1
Live procedure	6
dV-trainer Mimic Technologies	5 (8%)
da Vinci Surgical Skills simulator (dVSS)	12 (20%)
Other Robotic Training modalities	8 (13%)

N.B. Other Robotic Training modalities include: RobotiX Mentor, Senhance Surgical System, ProMIS and AdLap

### Assessment tools

**Fig. 3:**
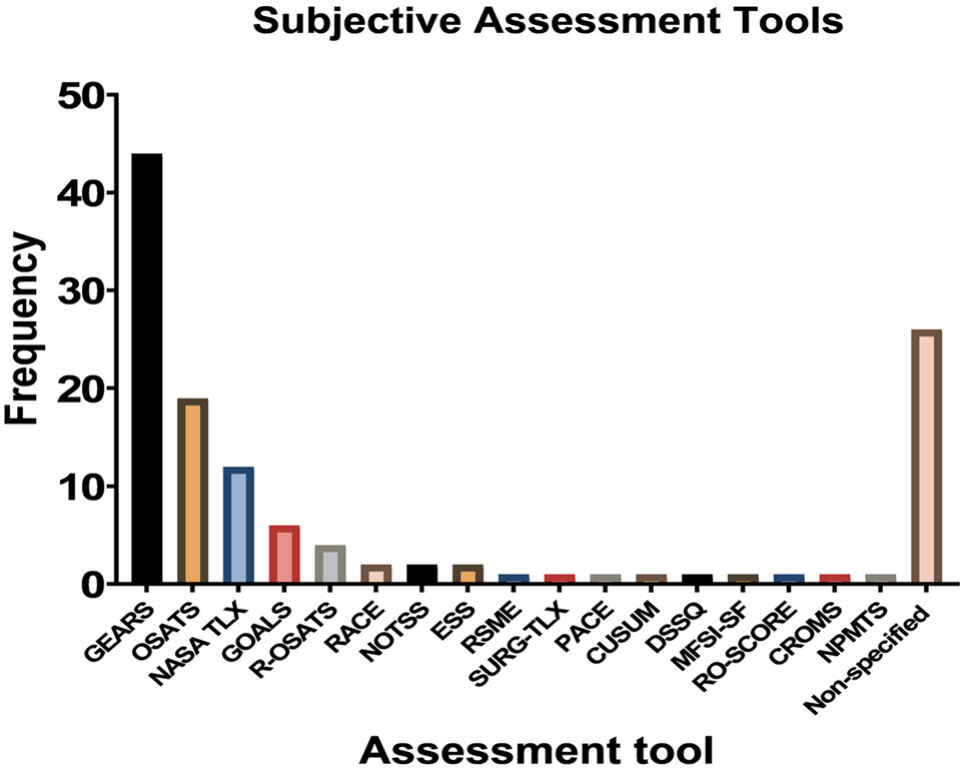
Subjective assessment tools. *N.B. GEARS* Global Assessment of Robotic Skills, *OSATS* Objective Structured Assessment of Technical Skill, *NASA TLX* Task Load Index, *GOALS* Global Operative Assessment of Laparoscopic Skills, *R-OSATS* Robotic—Objective Structured Assessment of Technical Skill, *ESS* Epworth Sleepiness Scale, *NOTSS* Non-Technical Skills for Surgeons, *RACE* Robotic Anastomosis Competence Evaluation, *RSME* Rating Scale for Mental Effort, *SURG-TLX* Task Load Index, *PACE* Prostatectomy Assessment and Competency Evaluation, *ARCS* Assessment of Robotic Console Skills, *SARMS* Structured Assessment of Robotic Microsurgical Skills, *DSSQ* Dundee Stress State Questionnaire, *MFSI-SF* Multidimensional Fatigue Symptom Inventory—Short Form, *RO-SCORE* Ottawa Surgical Competency Operating Room Evaluation, *CROMS* Clinically Relevant Objective Metrics of Simulators, *NPMTS* Numeric Psychomotor Test Score, *NTSRS* Non-Technical Skills in Robotic Surgery Score, *ICARS* Interpersonal and Cognitive Assessment for Robotic Surgery

A total of 44 articles were identified where robotic skills were determined using the GEARS score (Global Assessment of Robotic Skills). Other validated assessment forms commonly used in robotic surgery training included Objective Structured Assessment of Technical Skill (OSATS) and Global Operative Assessment of Laparoscopic Skills (GOALS) (Fig. 3). Vaccaro et al. [23] evaluated the effectiveness of virtual reality training in improving surgical skill, which was measured using R-OSATS, a modified OSATS-score for robotic surgery. R-OSATS was included in other studies as well [24–26]. Non-technical skills such as leadership, communication, and situational awareness were also assessed during robotic surgery training. Two reports included Non-Technical Skills for Surgeons (NOTTS) [27, 28], while other studies measured non-technical skills using Interpersonal and Cognitive Assessment for Robotic Surgery (ICARS) and Non-Technical Skills in Robotic Surgery Score (NTSRS) [29].

Various subjective forms aimed to assess physical strain during robotic surgery. Overall, 12 articles measured workload using the validated NASA Task Load Index (TLX). Moore et al. [30] compared the workload between robotic-assisted tasks and laparoscopic tasks using the Rating Scale for Mental Effort (RSME) and the Surgery Task Load Index (SURG-TLX). Two articles focused on the effect of fatigue on robotic skills, where levels of fatigue were assessed using the Epworth Sleepiness Scale [31, 32].

**Fig. 4:**
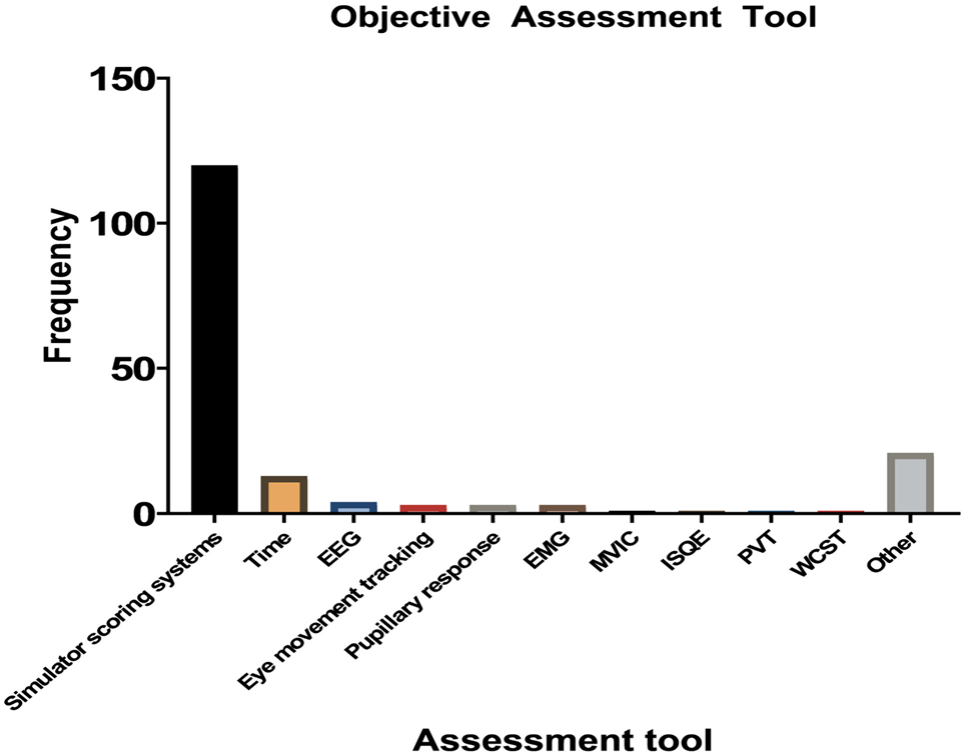
Objective assessment tools. *EMG* eye movements, *MVIC* maximum voluntary isometric muscle contractions, *ISQE* Indirect Sealing Quality Evaluation, *PVT* Psychomotor Vigilance Task, *WCST* Wisconsin Card Sorting Test

A total of 120 articles were identified in which robotic skills were determined using a built-in scoring system that collects objective data from simulators (Fig. 4). These scoring systems were mostly available for the dVSS and the dV-trainer. The scoring systems calculated a cumulative total score based on multiple parameters such as time, motion, work space, collisions, excessive force, and instruments out of field. Total completion time was used as an objective parameter in all 120 articles, and in 13 of those (11%), the time to complete the task was the only objective outcome measured.

Multiple studies focused on cognitive metrics during robotic training. Wu et al. [33] determined the correlation between cognitive and behavioral metrics with skill level. In this study, EEG and pupillary response were indicators of cognition and workload. Other reports measured the pupil diameter as well [34, 35].

Out of the total 209 studies, 47 studies (23%) used subjective assessment methods such as assessment forms, while 102 articles (49%) used objective assessment tools (Supplemental File A, Fig. A2). 60 studies (28%) used a combination of subjective and objective assessment methods. The majority of the objective assessment methods consisted of scoring systems inherent to the specific training modality in question.

### Timeline of training modalities and assessment methods

From 2005 to 2014, a majority of studies included a virtual or digital training modality. These studies primarily focused on initial learning curves, with the dominant assessment method being the dVSS built-in scoring system. Additionally, some initial studies involving dry lab were conducted, where performance was assessed using the OSATS form.

From 2014 onwards, a shifts was observed in the studies, with a primarily focus on analyzing the transfer of virtual and dry lab skills to the wet lab and operating room. Furthermore, training was not only limited solely to virtual or digital training, but a majority of the studies incorporated a second more invasive modality such as animal tissue, human cadaver labs, or direct practice in the operating room. Increasingly, studies incorporated an additional assessment form alongside the dVSS to assess both general procedural skills and procedure-specific skills. The dominant assessment form in this regard was the GEARS assessment form.

### Transferability of skill

In total, 45 studies primarily focused on the transferability of simulation skills to the operating room. In 42 (93%) of the studies, the initial training and assessment consisted of virtual training on the dVSS with dVSS metrics as assessment, and dry lab sessions with GEARS assessment. Performance in the operating room was assessed either in real-time or with videos using the GEARS form. The control group received no simulation training. In 37 (82%) of the studies, simulation performance was correlated with better performance in the operating room.

## Discussion

This systematic review provides a comprehensive overview of training in robotic-assisted surgery and the corresponding assessment methods. Since the da Vinci Surgical System has been the most widely used robotic surgery system in the past years, training has primarily focused on this system, particularly through dry lab training using suture pads and artificial tissue. Dry lab training offers a high-fidelity simulation environment that enables trainees to practice and hone their technical skills in a safe and controlled setting [28, 36, 37]. Box trainers enable the training of advanced technical skills with visual tissue interaction. There is an absence of haptic feedback, but this is compensated for by the visual component of instrument and tissue interaction forces. Through visual cues and tissue manipulation, a trainee learns to deal with the absence of haptic feedback. In addition, dry lab training with bio tissue and 3D-printed tissue models can also be used in conjunction with objective assessment methods, such as force measurements. This can provide valuable information about a trainee’s ability to handle and manipulate tissue during surgery, thus enhancing assessment accuracy. Assessment in box training commonly involves subjective assessment forms like the validated GEARS and OSATS forms [38–40]. While these forms have been validated by experts and have various modified versions, their generalizability is limited. Continuous validation is necessary for new trainings tasks or procedures, and assessment is dependent on the presence or post hoc evaluation of an expert, and interrater variability has to be taken into account.

The second most commonly used training modality is the da Vinci Skills Simulator (dVSS). Virtual reality (VR) training allows improvement in basic technical skills, procedural training tasks, and familiarization with the user interface, controls, and camera [41–43]]. VR training provides a convenient and cost-effective way to train and assess surgical skills. Similar to dry lab training, a challenge of VR training is the absence of haptic feedback. Current technology lacks the ability to simulate realistic virtual tissue and instrument interaction forces. As a results, the feedback and assessment incorporated in VR trainers are limited to parameters like time, motion, and task-specific errors. Another challenge of VR training is the limited availability of the master console, requiring trainees to train outside of regular working hours in the hospital. With the dVSS being a major training modality, the dVSS assessment score is also the most commonly used feedback and assessment method.

Ideally, one training modality should not replace the other due to their inherent drawbacks, but instead they should complement each other. A curriculum ideally comprises a step-by-step methodology, progressively incorporating more invasive modalities and increasing difficulty. For instance, basic training could commence with theory, including e-learning, narrated videos, or presentations. Subsequently, system training could be conducted using the digital training platform of the surgical robot to familiarize the trainee with the user interface, controls, cameras, and technicalities. Following this, the initial technical skills such as bimanual dexterity, depth perception, and tissue manipulation can be honed in the digital trainer. Upon achieving validated benchmarks, dry lab can commence. Here, objective force and motion sensors provide feedback on technical skills, while assessment tools such as GEARS for general skills and modified assessment forms for specific procedures offer personalized feedback to trainees.

Next, advanced training is conducted to train both technical and procedural skills on ex-vivo animal tissue or live animals, again with feedback provided through GEARS. Furthermore, procedural skills could also be trained and assessed with human cadaver labs. A proctored procedure can then follow, and after doing a number of cases, a learning session can be scheduled to review the experiences of the robot learning curve and evaluate tips and tricks with peers.

Another observation is the limited variety in study protocols and methods. A significant number of studies compared VR training to no training and assessed performance using subjective forms in VR or box training. However, what is lacking is a comparison of different training methods. Future training modalities should incorporate objective parameters for assessing skill acquisition and reaching preset proficiency levels. Objective feedback should not only focus on time and motion but also on tissue manipulation, handling, interaction forces on the tissue, and specific quality assessment like sutures. Furthermore, to optimize training effectiveness and efficiency, prediction models and machine learning could be used to predict the necessary training load for each trainee. Objective assessment would also enable the comparison of learning curves on different robotic-assisted surgery systems. As the number of robotic surgery systems increases, a multi-RAS platform for training and assessment will be essential for training and evaluating proficiency across different robotic systems.

## Conclusion

This systematic review provides an overview of training modalities and assessment methods in robotic-assisted surgery. Dry lab training on the da Vinci Surgical System and virtual training on the da Vinci Skills Simulator are the most commonly used approaches. However, focused training on tissue handling, manipulation, and force interaction is lacking, considering the absence of haptic feedback. Future research should focus on the development of universal objective assessment and feedback methods to address these limitations as the field continues to evolve.

### Supplementary Information

Below is the link to the electronic supplementary material.

Supplementary file1 (DOCX 232 KB)
