# Supplementary material for: Training in robotic-assisted surgery: a systematic review of training modalities and objective and subjective assessment methods
Source: Surg Endosc. 2024 May 30;38(7):3547–55. doi: 10.1007/s00464-024-10915-7 (PMC11219449; doi:10.1007/s00464-024-10915-7)
Supplement: Supplementary file 1 — Supplementary file1 (DOCX 232 KB) [file 464_2024_10915_MOESM1_ESM.docx]

**Supplemental File A**

**Table A1**

Search strategy Pubmed

| #1 "robotic surgery"[Title/Abstract] OR "robot assisted surgery"[Title/Abstract] OR "RAMIS"[Title/Abstract] OR "robotic surgical system"[Title/Abstract] OR "robotic surgical procedure"[Title/Abstract] OR "robotic surgical procedures"[MeSH Terms]  #2 "objective"[Title/Abstract] OR "subjective"[Title/Abstract] OR "assessment"[Title/Abstract] OR "subjective assessment"[Title/Abstract] OR "objective assessment"[Title/Abstract]  #3 "training"[Title/Abstract] OR "curriculum"[Title/Abstract] OR "curricula"[Title/Abstract] OR "VR"[Title/Abstract] OR "vr training"[Title/Abstract] OR "virtual reality"[Title/Abstract] OR "hands on training"[Title/Abstract] OR "proctoring"[Title/Abstract] OR "box training"[Title/Abstract] OR "dry lab"[Title/Abstract] OR "cadaver"[Title/Abstract] OR "mentoring"[Title/Abstract] |
| --- |

**Table A2**

Search strategy EMBASE

| ('robotic surgery':ab,ti OR 'robot assisted surgery':ab,ti OR ramis:ab,ti OR 'robotic surgical system':ab,ti OR 'robotic surgical procedure*':ab,ti) AND (objective:ab,ti OR subjective:ab,ti OR assessment:ab,ti OR 'subjective assessment':ab,ti OR 'objective assessment':ab,ti) AND (training:ab,ti OR curriculum:ab,ti OR curricula:ab,ti OR vr:ab,ti OR 'vr training':ab,ti OR 'virtual reality':ab,ti OR 'hands on':ab,ti OR proctoring:ab,ti OR 'box training':ab,ti OR 'dry lab':ab,ti OR cadaver:ab,ti OR mentoring:ab,ti) |
| --- |

**Table A3**

Search strategy Cochrane

| robotic surgery OR robot assisted surgery OR RAMIS OR robotic surgical procedures OR robotic surgical system in Title Abstract Keyword AND objective OR subjective OR assessment OR subjective assessment OR objective assessment in Title Abstract Keyword AND training OR curriculum OR curricula OR VR OR vr training OR virtual reality OR hands on OR proctoring OR box training OR dry lab OR cadaver OR mentoring in Title Abstract Keyword – (Word variations have been searched) |
| --- |

**Figure A1**

Basic and advanced course


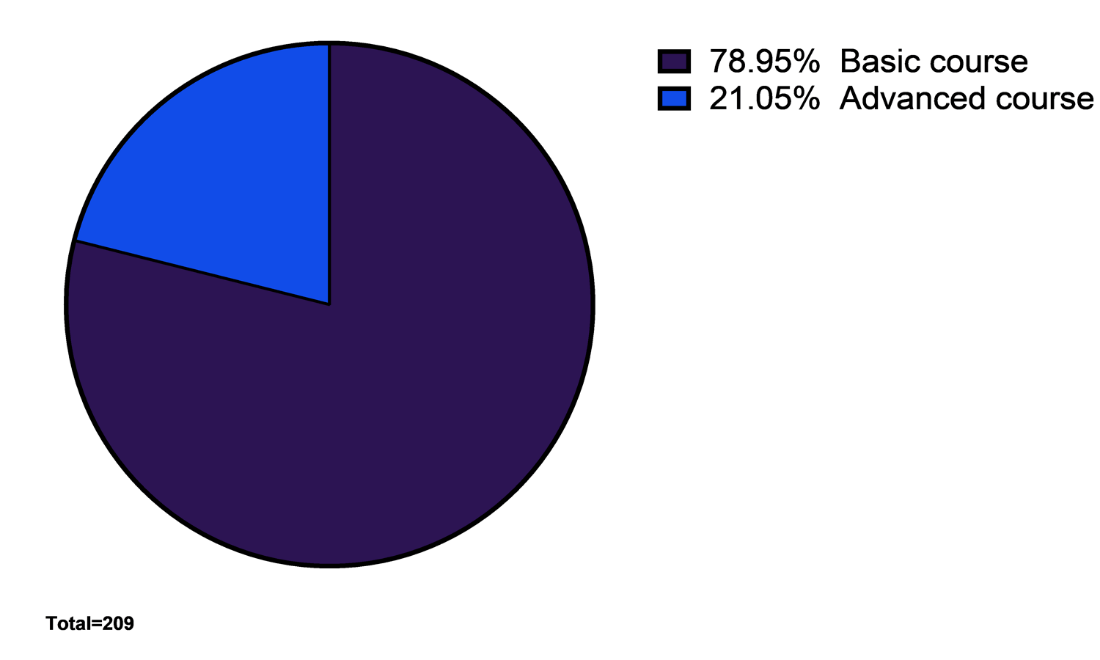


**Figure A2**

Objective and subjective assessment in robotic surgery training

**
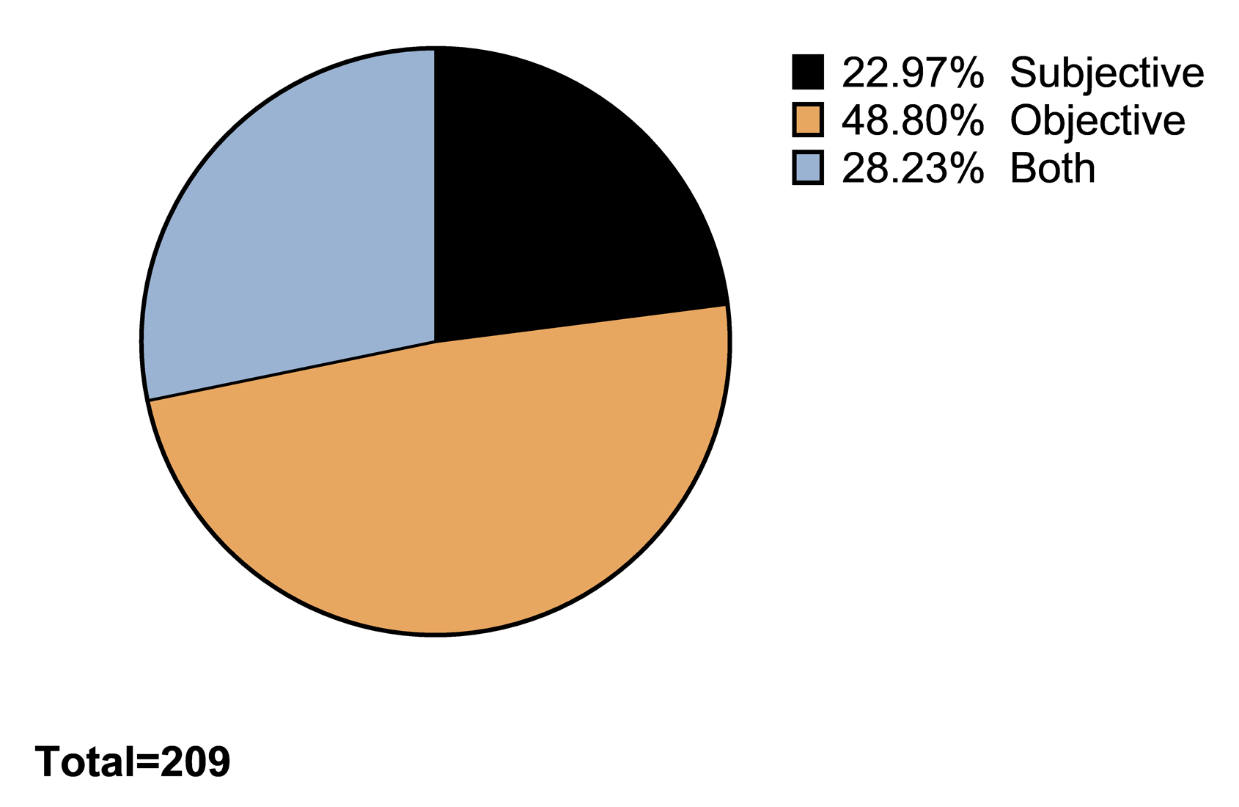
**
